# Verification of daily dose recalculation accuracy for an adaptive radiation therapy monitoring tool in helical tomotherapy for nasopharyngeal carcinoma

**DOI:** 10.1002/acm2.14601

**Published:** 2024-12-03

**Authors:** Yawitta Maneepan, Anirut Watcharawipha, Imjai Chitapanarux, Somsak Wanwilairat, Wannapha Nobnop

**Affiliations:** ^1^ Medical Physics Program, Department of Radiology, Faculty of Medicine Chiang Mai University Chiang Mai Thailand; ^2^ The Division of Radiation Oncology, Department of Radiology, Faculty of Medicine Chiang Mai University Chiang Mai Thailand

**Keywords:** adaptive radiation therapy (ART), dose monitoring, dose recalculation, helical tomotherapy, PreciseART

## Abstract

**Purpose:**

PreciseART, an adaptive radiation therapy (ART) software for helical tomotherapy (HT), was integrated into the Precision treatment planning system (Accuray, Inc., Sunnyvale, CA). It supports automatic monitoring of dose variations to both the target and organs at risk (OARs) throughout the treatment course. This study aims to verify the accuracy of PreciseART's automatic dose recalculation and assess the effectiveness of its notification function.

**Methods:**

The Radixact X9's kVCT image‐guided system (ClearRT) was used to acquire daily images for dose recalculations. We assessed the accuracy of PreciseART's automatic dose recalculation by comparing it with the treatment planning system (TPS) recalculation for the PTV70, spinal cord, and bilateral parotid glands. We also evaluated the efficacy of its notification function by comparing each dose metric for each notification color to TPS recalculation, assessing its role as a trigger tool.

**Results:**

In the phantom study, dosimetric analysis indicated no statistically significant differences between TPS and PreciseART recalculations (*p* > 0.05), with dose variations under 1.5%. Similarly, in the patient study (*n* = 10), no significant dosimetric discrepancies were found (*p* > 0.05), with a maximum variation of 2.3%. The notification system performed effectively, providing accurate notifications in accordance with predefined dose criteria.

**Conclusions:**

PreciseART's daily dose recalculation demonstrated good agreement with TPS recalculation, and its notification function is effective for identifying dose threshold compliance, supporting its use in clinical ART.

## 1 INTRODUCTION

Nasopharyngeal carcinoma (NPC) is a common cancer in Southeast Asia that exhibits an irregular shape and its proximity to organs at risk (OARs) such as the brainstem, spinal cord, and parotid glands.^1^ Consequently, intensity‐modulated radiotherapy (IMRT) is an appropriate treatment technique for NPC, as it produces high‐dose gradients that enhance target coverage while sparing OARs.^2^ However, anatomical changes during treatment can affect the dose received by both the target and OARs, potentially increasing the risks of complications and recurrence.^2^ In order to address these changes, adaptive radiation therapy (ART) creates a new treatment plan that takes anatomical changes into consideration.^3^

Although ART offers significant benefits for head and neck cancers, only 55% of treatment centers currently implement it.^4^ The hybrid plan recalculates dose based on updated imaging to assess anatomical changes, helping physicians decide if adaptive replanning is needed. However, creating a hybrid plan is challenging and resource‐intensive, contributing to the fact that only 10% of centers have predefined action levels, while 45% prefer non‐protocol ART due to limited material and human resources.^4^ Recently, commercial software such as RTTapp (SegAna, Orlando, FL), PERFRACTION (Sun Nuclear Corp., Melbourne, FL), and PreciseART (Accuray, Inc., Sunnyvale, CA) has become available, offering tools to monitor and evaluate dosimetric changes with reduced manual effort. These software programs could support treatment centers in advancing toward an individualized ART implementation, improving treatments more precisely to patient‐specific changes.

Helical tomotherapy (HT), a ring‐based IMRT, has proven effective in treating NPC, with improved mean conformity and homogeneity indices.^1^, ^5^ The original image‐guided system is the megavoltage computed tomography (MVCT). Recently, a new image‐guide system called “ClearRT” (Accuray, Inc., Sunnyvale, CA) has been introduced. ClearRT is a helical fan‐beam kVCT imaging system that provides image quality comparable to CT with better clarity than MVCT.^6^ ClearRT's high‐quality images and detailed anatomical visualization make it a promising tool for dose calculation, potentially improving ART accuracy.^7^ To support ART for HT, PreciseART—an automated ART‐supporting software program integrated into the Precision TPS—enables daily dose recalculation, daily dose monitoring, and daily structural contour deformation. The dose recalculation is done on merged images that combine the planning CT and the daily IGRT, addressing areas that the IGRT is unable to cover because of its limited scan range and FOV. This process also interpolates slice thickness to match the planning CT resolution. The computation includes both the CT number‐to‐physical‐density table and the planned sinogram, utilizing the same dose calculation algorithm as the treatment planning system (TPS). Additionally, PreciseART allows users to set dosimetric thresholds for each structure for being a trigger tool, with notifications in red, yellow, or green indicating dosimetric changes.^8^

Tools like PreciseART, which offer automatic monitoring of dosimetric changes, could streamline ART implementation. The software marks color notifications to represent both scaled and projected dose reports. The projected dose reflects a deformable dose accumulation (DDA) estimate for the end of treatment. The accuracy of the projected dose depends on three critical factors: the accuracy of daily dose calculation, the quality of deformable image registration (DIR) between daily images, and the accuracy of dose transformation. Together, these factors determine the overall reliability of the projected dose.^9^ García‐Alvarez et al.^10^ investigated the uncertainty bounds of PreciseART's DDA and recommended using the projected dose as a trigger tool after the mid‐treatment course, as it provides results closer to the end of treatment. However, its accuracy tends to decrease with significant anatomical changes, which can undermine the reliability of the DDA. Additionally, performing quality assurance (QA) on the DDA process remains complex.

However, the previous study has not clarified the accuracy of daily dose calculations, which are the basis for scaled and projected doses, that are essential for providing reliable dose information in ART decision‐making. Therefore, the primary aim of this study is to verify the accuracy of PreciseART's daily dose recalculation by comparing it with the TPS recalculation in both phantom and patient studies. Additionally, using the scaled dose as a trigger tool aligns with the hybrid plan approach, a fundamental method for evaluating dosimetric changes. Consequently, the second aim is to assess the performance of PreciseART as a trigger tool in monitoring dose changes based on the scaled dose.

## 2 MATERIALS AND METHODS

### 2.1 Image acquisition and treatment planning

CT images of an anthropomorphic phantom (RANDO Woman Phantom, Alderson Research Laboratories, Stanford, CT) and 10 NPC patients were acquired using a CT simulator (Somatom Definition AS; Siemens, Germany) according to standard protocols: 120 kVp, automatic tube current, 550 mm FOV, and 2 mm slice thickness from vertex to carina. These images were exported to the TPS.

Helical treatment plans were created for delivery by the Radixact X9 with the ClearRT imaging system. The prescribed dose was 70 Gy at 2.12 Gy per fraction to the high‐risk PTV (PTV70), with simultaneous boosts to the intermediate‐risk PTV (PTV59.4) at 1.8 Gy per fraction and low‐risk PTV (PTV54) at 1.64 Gy per fraction, over 33 fractions. All plans were approved according to ICRU83 and RTOG protocols, as detailed in Table 1.

**TABLE 1:** The dose constraint criteria for plan evaluation in Precision TPS and the predefined notification levels in PreciseART during treatment.

	Dose‐volume constraints				
Volume of interest (VOI)	Dosimetric parameters (Gy)	Plan evaluation (green) (Gy)	Tolerance level (yellow) (Gy)	Action level (red) (Gy)	Scaled dose to single fraction (Gy)
PTV70	*D*_98%_	≥66.5	64.4–66.5	<64.4	<1.95
	*D*_50%_	=70	67–70	<67	<2.03
	*D*_2%_	≤74.9	74.9–77	>77	>2.33
Parotid glands (each)	*D*_50%_	<30	30–35	>35	>1.06
Spinal cord	*D*_2%_	<45	–	>45	>1.36

A preliminary study was conducted to select the appropriate ClearRT imaging protocol for dose calculation. The protocol—Thorax Anatomy, Medium body size, 440 mm FOV, and Normal scan mode—showed no statistically significant dose differences compared to CT simulation, with dose variations ranging from −0.5% to 0.98%, consistent with the findings of Yang et al.^7^ Therefore, this ClearRT protocol was applied consistently across all cases in this study.

### 2.2 Intensity value to density table (IVDT) of ClearRT images for dose calculation

Half of the TomoPhantom (Cheese Phantom, Accuray, Inc., Sunnyvale, CA), containing multiple density plugs and positioned on a 2 cm Virtual Water slab base, was scanned using the ClearRT system. The scanning parameters were set to thorax anatomy, medium body size, 440 mm FOV, and normal scan mode. The images were exported to MIM Maestro software version 7.3.3 (MIM Software Inc., Cleveland, OH) for analysis. Subsequently, the mean HU values for each density plug were input into the Precision TPS to generate the IVDT for the ClearRT images.

### 2.3 The accuracy of dose calculation: A phantom study

**FIGURE 1:**
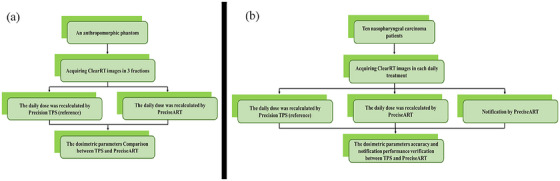
Study diagrams: (a) Phantom study diagram and (b) patient study diagram.

ClearRT images were acquired for both PreciseART and TPS recalculations. To verify PreciseART's dose recalculation accuracy, dose differences between TPS and PreciseART results were compared under identical conditions. The comparison results were presented in terms of DVH metrics, including *D*_98%_, *D*_50%_, and *D*_2%_ for the PTV70; as well as *D*_2%_ for the spinal cord; and *D*_50%_ for bilateral parotid glands; see process diagram in Figure 1a.

### 2.4 Daily dose monitoring and verification: Patient study

**FIGURE 2:**
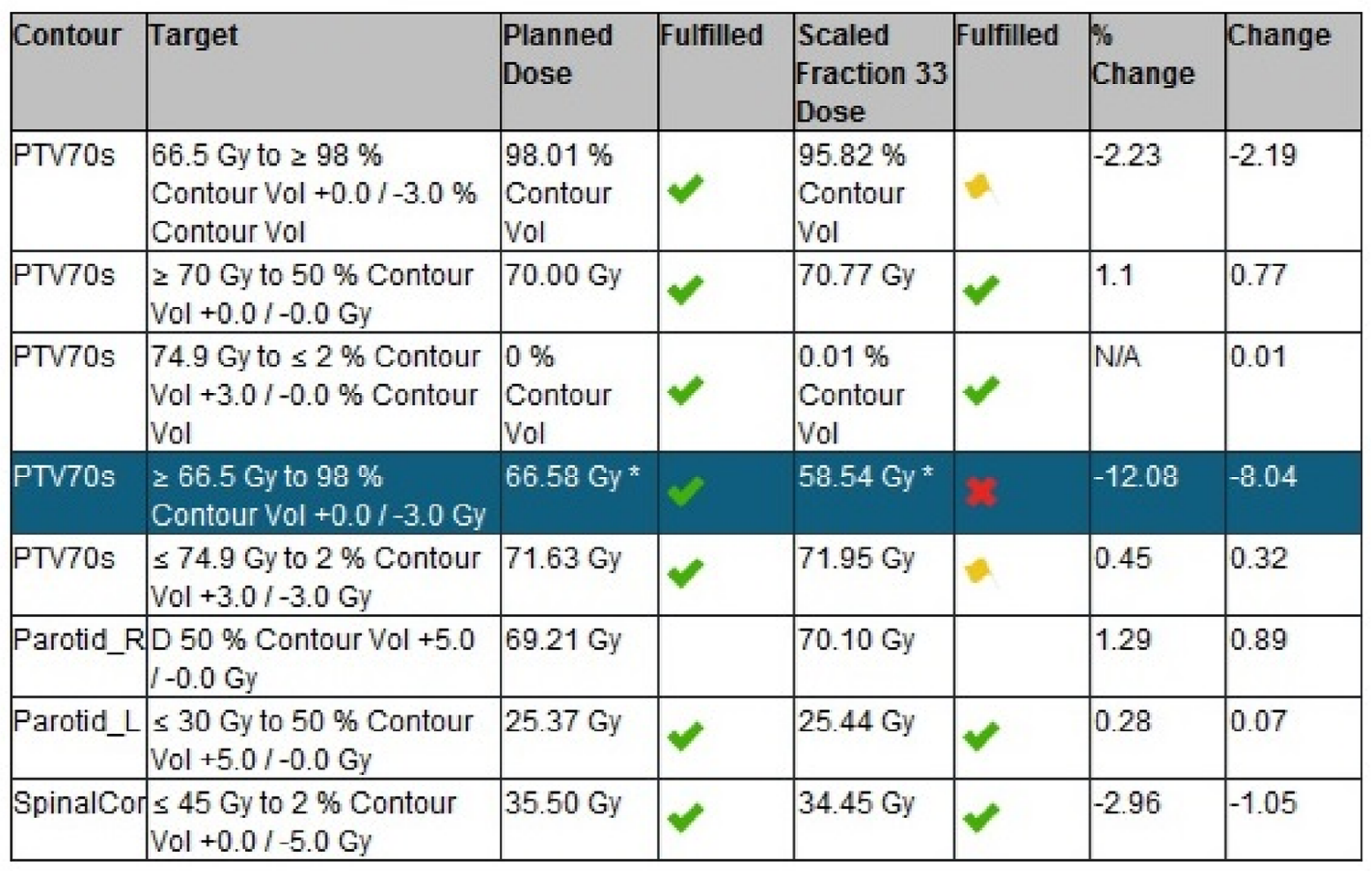
Example of PreciseART results with notifications triggered by scaled dose.

The patient study evaluated the dose recalculation accuracy and trigger tool performance of PreciseART (see process diagram in Figure 1b). Notification color levels for each patient were set to a predetermined threshold for the spinal cord, parotid glands, and PTV70 according to the criteria in Table 1. Figure 2 shows an example report with notification signs based on scaled doses.

For dose recalculation verification, instances where the same DVH metric triggered with red notification for at least 3 consecutive days were assessed. The recalculated plan was created in TPS using the daily ClearRT images associated with the red notification to verify the accuracy of the PreciseART. The evaluation included *D*_98%_, *D*_50%_, and *D*_2%_ for the PTV70; *D*_2%_ for the spinal cord; and *D*_50%_ for bilateral parotid glands. For trigger tool performance evaluation, the mean DVH metrics were compared between TPS and PreciseART results for each notification level: green, yellow, and red. The study was reviewed and approved by the Board of Faculty of Medicine at Chiang Mai University (study code RAD‐2566‐0577/research ID: 0577)

### 2.5 Dosimetric comparison and statistical analysis

The dose differences in *D*_98%_, *D*_50%_, and *D*_2%_ of the PTV70; *D*_2%_ for the spinal cord; and *D*_50%_ for bilateral parotid glands between PreciseART and TPS results were analyzed in both phantom and patient studies. DVH metrics for the PTV70, spinal cord, and bilateral parotid glands were summarized as mean ± SD. The normality of the DVH metrics was tested using the Shapiro‐Wilk or Kolmogorov‐Smirnov tests. Paired dosimetric differences were analyzed using the Wilcoxon signed‐rank test (non‐normal distributions) or paired *t*‐test (normal distributions), with significance set at *p* < 0.05. Statistical analyses were conducted using IBM SPSS 26.0 (IBM Corp., Armonk, NY).

## 3 RESULTS

### 3.1 The accuracy of dose calculation: A phantom study

**FIGURE 3:**
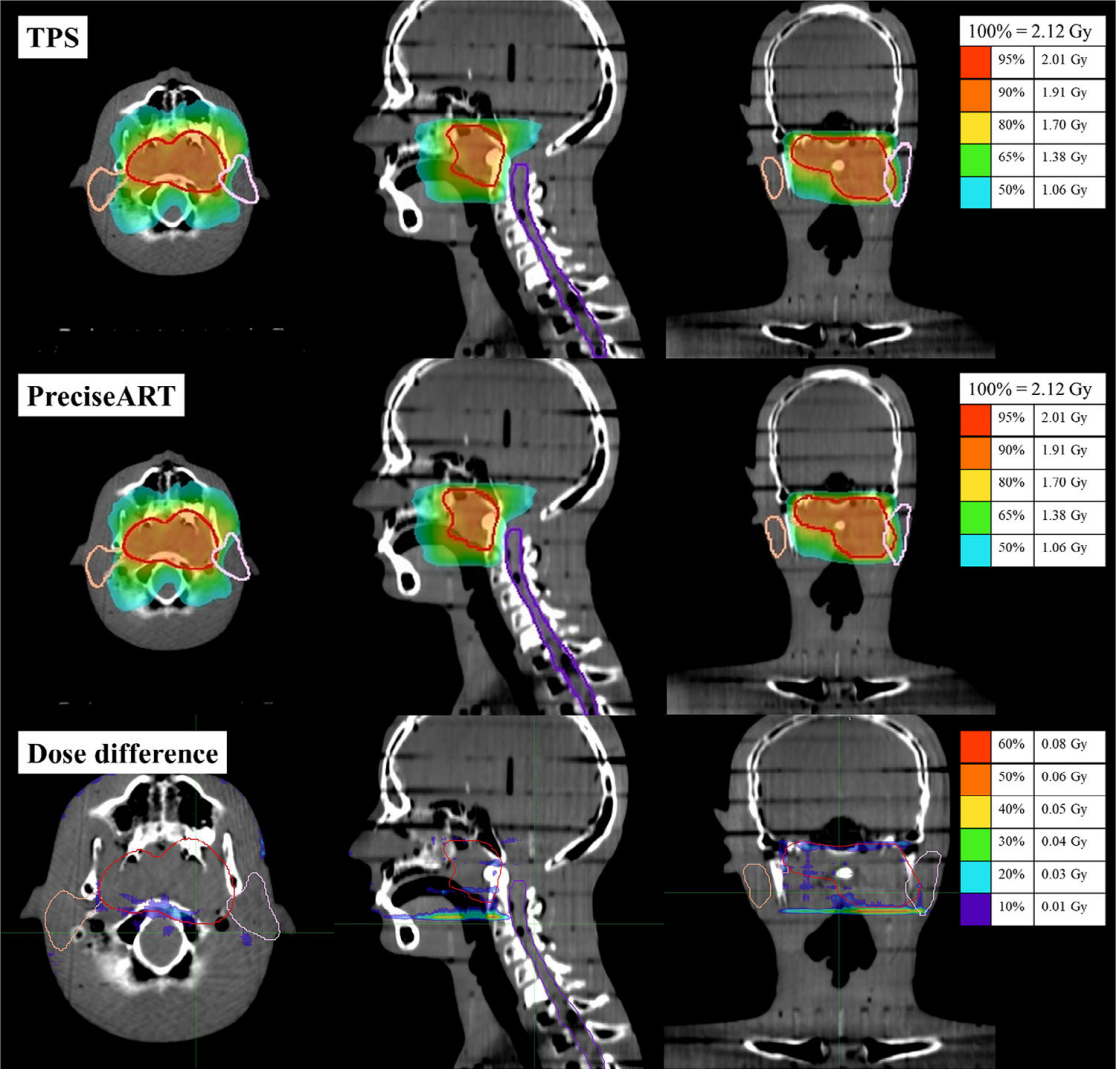
The dose distribution was recalculated for the phantom using TPS (top) and PreciseART (middle). The dose difference between TPS and PreciseART is presented on the ClearRT images (bottom).

**FIGURE 4:**
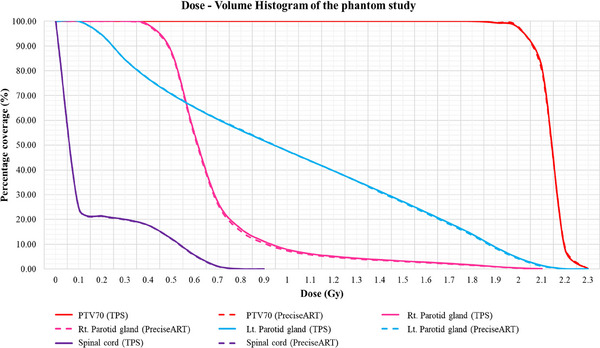
DVH of PTV70 (red), right parotid (pink), left parotid (blue), and spinal cord (purple) for the phantom study. The solid line represents TPS results, and the dashed line represents PreciseART results.

The phantom was irradiated three times, with ClearRT images acquired for each session. The phantom study demonstrated no significant difference between TPS and PreciseART recalculations (*p* > 0.05). The percentage dose differences were less than 1% for the target and OARs, except for the left parotid gland, which showed a 1.41% difference, as summarized in Table 2. These findings demonstrate good agreement between TPS and PreciseART recalculation results for the target and OARs, as illustrated in Figures 3 and 4.

**TABLE 2:** DVH metric differences between TPS and PreciseART recalculations in the phantom study.

Volume of interest (VOI)	Dosimetric parameters (Gy)	PreciseART (Gy)	TPS (Gy)	Δ Dose (Gy)	% Δ Dose (%)	Wilcoxon *p*‐value
PTV70	*D*_98%_	1.990 ± 0.00	1.990 ± 0.00	0.000 ± 0.00	0.00 ± 0.00	1.000
	*D*_50%_	2.160 ± 0.00	2.160 ± 0.00	0.000 ± 0.00	0.00 ± 0.00	1.000
	*D*_2%_	2.200 ± 0.00	2.210 ± 0.00	−0.010 ± 0.00	−0.45 ± 0.00	0.083
Right parotid	*D*_50%_	0.607 ± 0.01	0.610 ± 0.00	−0.003 ± 0.01	−0.55 ± 0.95	0.317
Left parotid	*D*_50%_	0.960 ± 0.01	0.947 ± 0.01	0.013 ± 0.01	1.41 ± 0.60	0.102
Spinal cord	*D*_2%_	0.670 ± 0.00	0.670 ± 0.00	0.000 ± 0.00	0.00 ± 0.00	1.000

*Note*: The dosimetric parameters of TPS recalculation were used as a reference.

### 3.2 Daily dose monitoring and verification: Patient study

**FIGURE 5:**
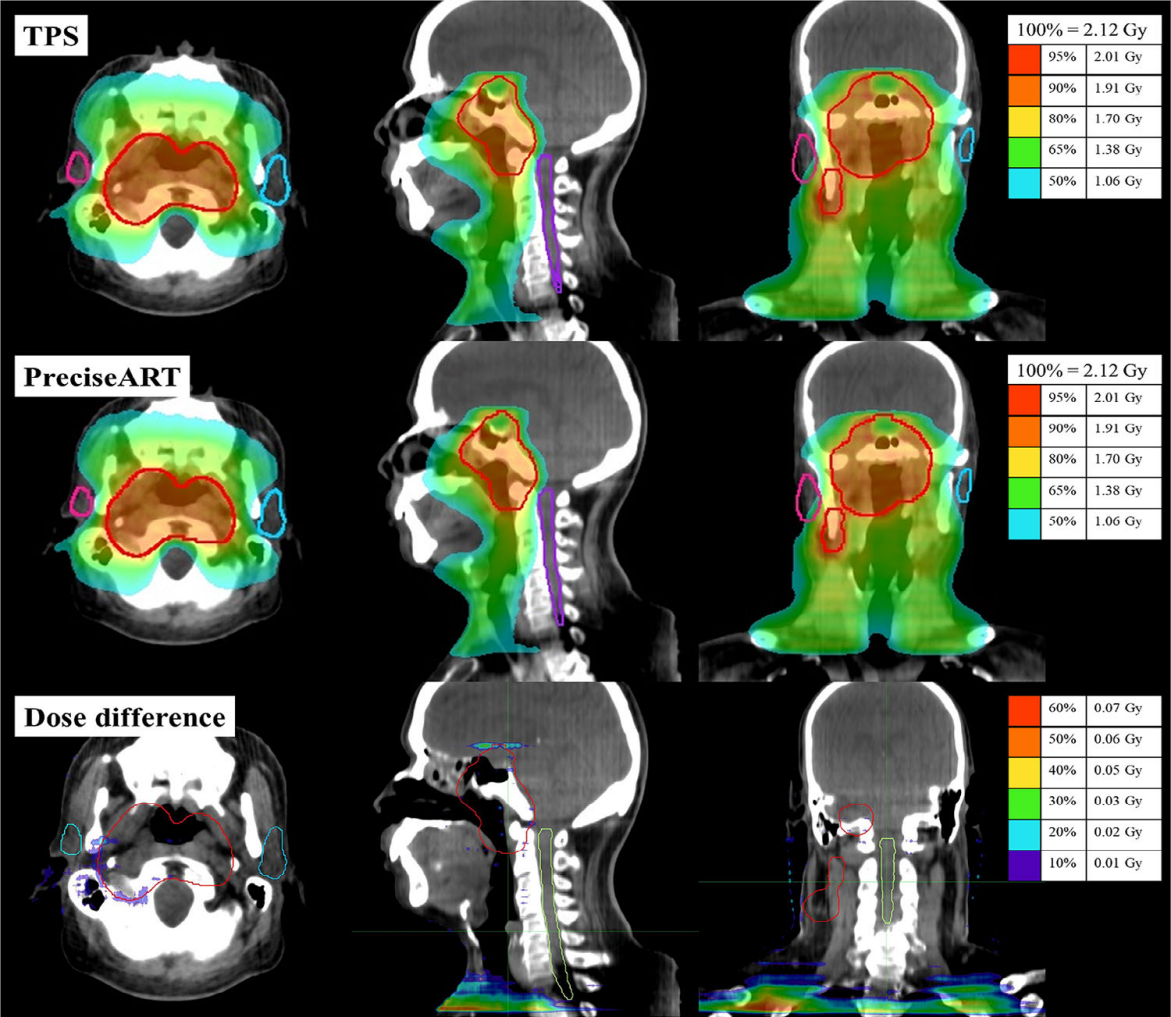
The dose distribution was recalculated for a sample patient using TPS (top) and PreciseART (middle). The dose difference between TPS and PreciseART is presented on the ClearRT images (bottom).

**FIGURE 6:**
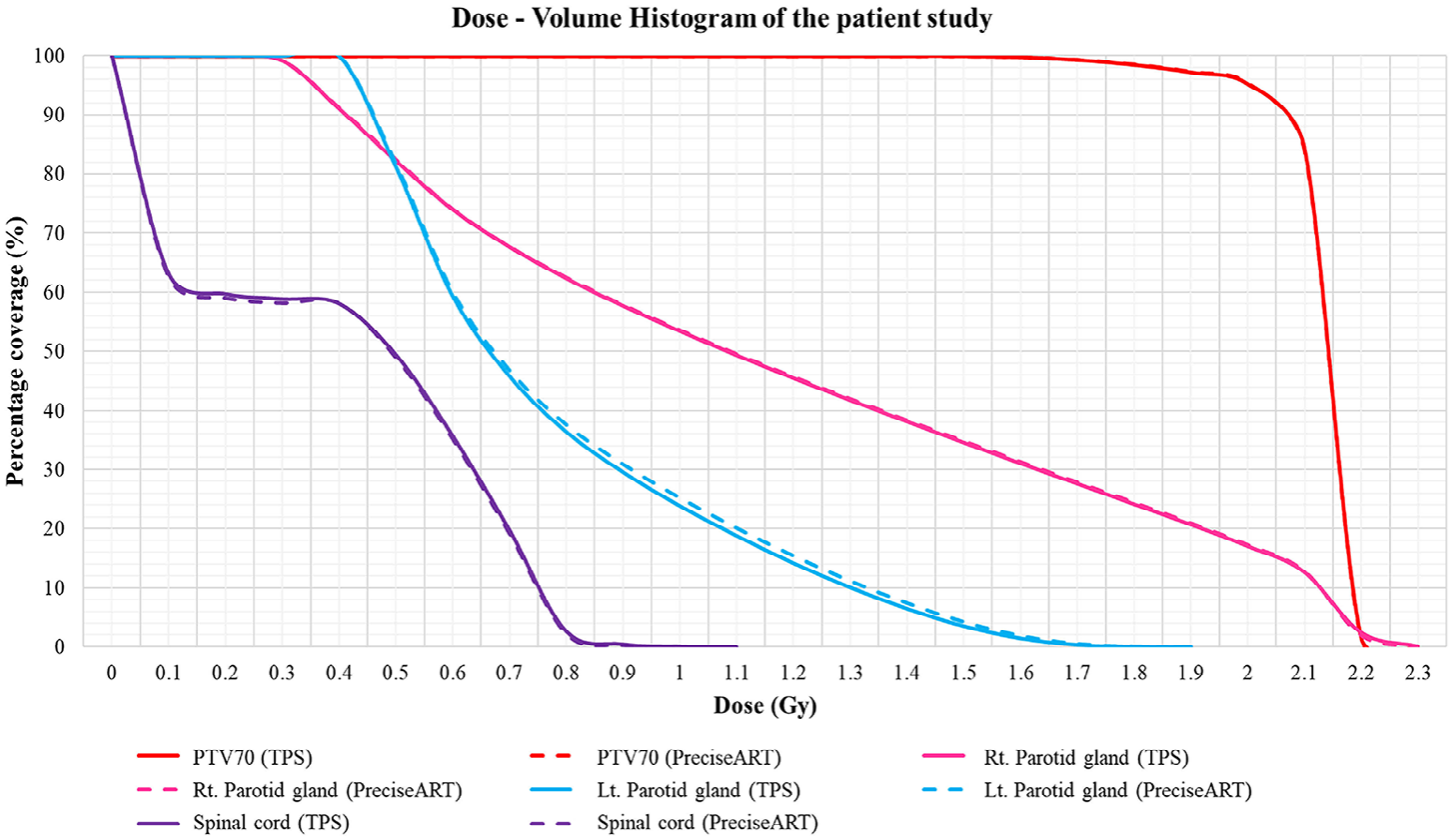
DVH of PTV70 (red), right parotid (pink), left parotid (blue), and spinal cord (purple) for the patient study. The solid line represents TPS results, and the dashed line represents PreciseART results.

Daily dose recalculation accuracy was verified using data that received a red notification for at least 3 consecutive days. Among the 10 patients, 8 received a red notification for at least 3 consecutive days, totaling 114 paired doses across all DVH metrics. Statistical analysis showed no significant dosimetric differences between TPS and PreciseART (*p* > 0.05). Dose differences were less than 1% for the PTV and left parotid gland, while the right parotid gland showed a 2.3% difference, as summarized in Table 3. The dose distribution, differences, and DVHs are displayed in Figures 5 and 6 for a sample patient whose treatment fraction received a red notification for the right parotid gland, while other parameters remained in the green.

**TABLE 3:** DVH metric differences between TPS and PreciseART recalculations for red notification in the patient study.

Volume of interest (VOI)	Dosimetric parameters (Gy)	PreciseART (Gy)	TPS (Gy)	Diff (% Diff)	*p*‐value
PTV70	*D*_98%_	1.856 ± 0.07	1.851 ± 0.08	0.004 ± 0.03 (0.27 ± 1.67)	0.434^a^
Right parotid	*D*_50%_	1.182 ± 0.10	1.159 ± 0.13	0.023 ± 0.06 (2.28 ± 4.80)	0.133^b^
Left parotid	*D*_50%_	1.216 ± 0.10	1.207 ± 0.10	0.009 ± 0.06 (0.84 ± 5.40)	0.634^a^

*Note*: The dosimetric parameters of TPS recalculation were used as a reference.

^a^ Wilcoxon sign rank test.

^b^ Paired‐samples *T* test.

For trigger tool performance evaluation, the TPS and PreciseART results were assessed across 1324 doses and 141 doses received green and yellow notifications, as shown in Table 4 (red notifications are shown in Table 3). Among the 141 doses with yellow notifications, the mean dosimetric differences ranged from −1.5 to 0.2 cGy (−1.03% to 0.44%) for the target and OARs. For the 1324 doses with green notifications, the mean dosimetric differences ranged from −2.2 to 0.2 cGy (−2.20% to 0.15%) for the target and OARs. The TPS and PreciseART results met the criteria for each notification, confirming that the PreciseART notification system was effective.

**TABLE 4:** DVH metric differences between TPS and PreciseART recalculations for yellow and green notifications in the patient study.

Alert sign	Volume of interest (VOI)	Dosimetric parameters (Gy)	PreciseART (Gy)	TPS (Gy)	Diff (% Diff)
Yellow	PTV70	*D*_98%_	1.980 ± 0.02	1.985 ± 0.03	−0.005 ± 0.02 (−0.24 ± 0.96)
	Right parotid	*D*_50%_	0.977 ± 0.06	0.975 ± 0.08	0.002 ± 0.05 (0.44 ± 5.00)
	Left parotid	*D*_50%_	0.956 ± 0.08	0.971 ± 0.11	−0.015 ± 0.07 (−1.03 ± 7.14)
Green	PTV70	*D*_98%_	2.052 ± 0.02	2.050 ± 0.02	0.002 ± 0.01 (0.08 ± 0.54)
		*D*_50%_	2.147 ± 0.01	2.154 ± 0.01	−0.008 ± 0.01 (−0.35 ± 0.37)
		*D*_2%_	2.205 ± 0.02	2.217 ± 0.03	−0.011 ± 0.02 (−0.50 ± 0.79)
	Right parotid	*D*_50%_	0.740 ± 0.12	0.762 ± 0.14	−0.022 ± 0.06 (−2.20 ± 6.94)
	Left parotid	*D*_50%_	0.746 ± 0.12	0.746 ± 0.12	0.000 ± 0.05 (0.15 ± 6.46)
	Spinal cord	D_2%_	0.979 ± 0.14	0.986 ± 0.14	−0.007 ± 0.02 (−0.64 ± 2.28)

*Note*: The dosimetric parameters of TPS recalculation were used as a reference.

**FIGURE 7:**
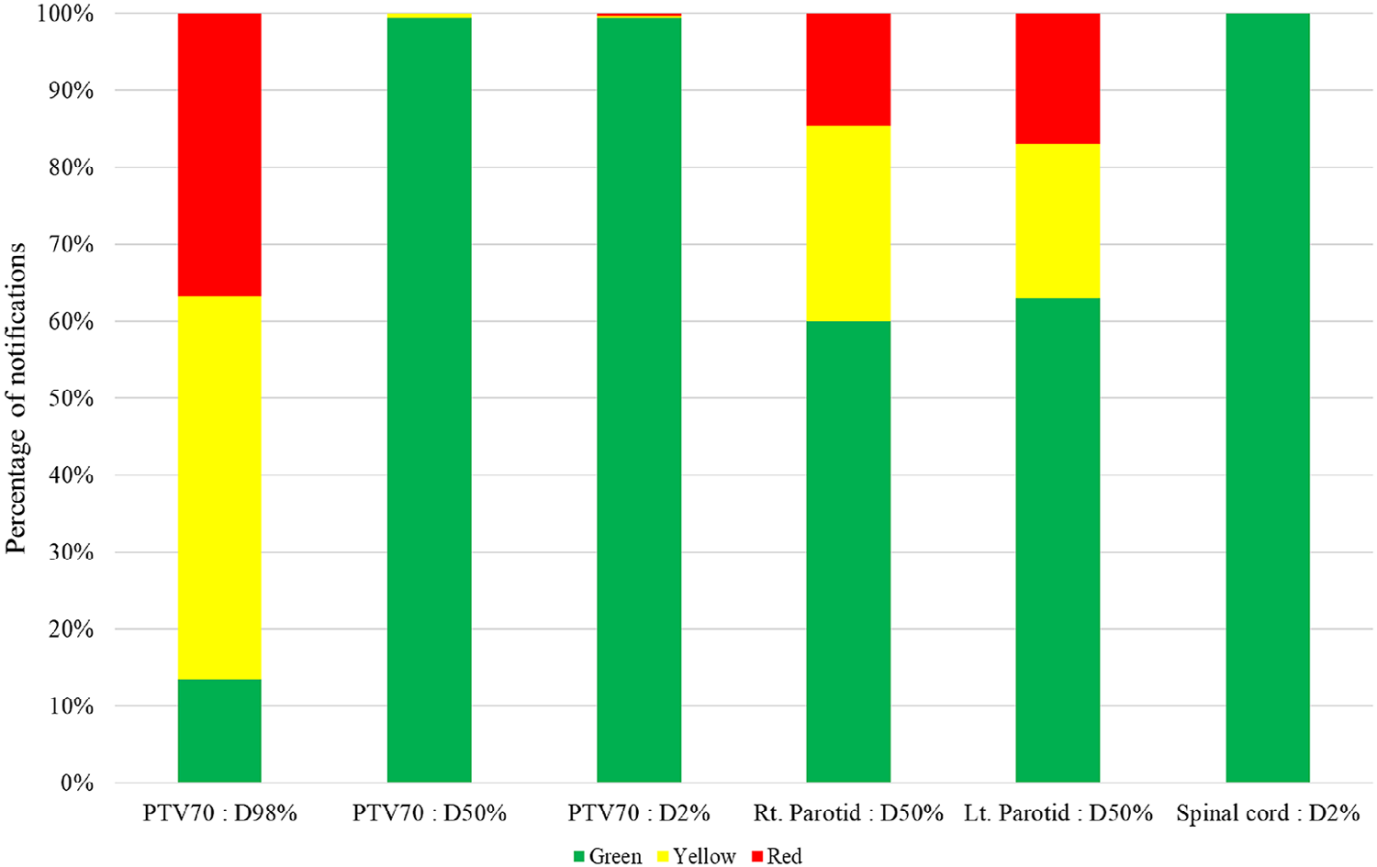
Frequency of notifications triggered in 10 NPC patients over 33 treatment fractions.

PreciseART was used as a trigger tool based on the scaled dose. Ten patients were observed over 33 fractions, with the number of patients monitored for each DVH metric varying due to action levels exceeding planning (see Table 5). The analysis showed that 77.16% of notifications were green, 13.17% were yellow, and 9.67% were red. The largest variations in DVH metrics were found in *D*_98%_ of PTV70, followed by the bilateral parotid glands, as shown in Figure 7. Eight of 10 patients received a red notification for at least 3 consecutive days, indicating dosimetric changes due to anatomical changes. Specifically, 71% of patients had red notifications for *D*_98%_ of PTV, with the first alert occurring on average at the 9^th^ fraction; 40% received red notifications for *D*_50%_ of the right parotid gland, with the first alert occurring on average at the 11^th^ fraction; and 50% received red notifications for *D*_50%_ of the left parotid gland, with the first alert occurring on average at the 16^th^ fraction. Based on these notifications, four patients underwent offline ART according to predefined adaptation criteria. However, only a single criterion was used to trigger plan adaptation in this study (see Table 1). In some cases, the original plan had lower doses for OARs, so even with anatomical changes, the doses remained below the action threshold. For example, in patients 2, 4, 8, and 9, the average left parotid dose in the original plan was 21.60 ± 2.57 Gy, increasing to 24.43 ± 2.76 Gy by the end of treatment, representing a 14.07 ± 17.53% increase. Therefore, using PreciseART as a trigger tool can allow for the adjustment of individualized criteria to improve plan adaptation for each patient.

**TABLE 5:** Number of patients for each investigated DVH metric and patients with red and yellow notifications for at least 3 consecutive days.

Volume of interest (VOI)	Dosimetric parameters	Total number of patients investigated	Number of patients alerted in red	Number of patients alerted in yellow
PTV70	*D*_98%_	7	5	1
	*D*_50%_	10	−	−
	*D*_2%_	10	−	−
Right parotid	*D*_50%_	5	2	1
Left parotid	*D*_50%_	10	5	1
Spinal cord	*D*_2%_	10	−	−

## 4 DISCUSSION

This study aims to verify the accuracy of dose recalculation and the efficiency of PreciseART as a trigger tool. Results from the phantom study indicate that PreciseART aligns with TPS results, as shown in Table 2 and Figures 2 and 3, with mean differences within 1.5% when no roll correction is applied during treatment. These findings confirm that PreciseART's automated process for generating merged images, selecting matched IVDT, and applying the planned sinogram functions effectively, using the same dose calculation algorithm as TPS. The minor differences observed in the phantom study are mainly due to variations in the FOV and slice thickness between the merged and ClearRT images, which affect the dose calculation grid size and volume in contoured structures.

PreciseART recalculates dose distribution using merged images that combine image‐guided (ClearRT or MVCT) and planning CT images, extending coverage to areas not included in IGRT. This process not only fills the images but also interpolates slice thickness to match the planning CT. Martin et al.^11^ studied treatment plans with varying MVCT slice thicknesses and merged images, finding that merged images achieved closer alignment with planned doses than MVCT alone, due to differences in calculation voxel size. Zhu et al.^12^ examined MVCT acquisition pitches and reconstruction intervals for dose calculation, reporting no significant differences even with intervals ranging from 1 to 6 mm. Other studies have similarly reported minimal dose differences with varying MVCT slice thicknesses.^13^, ^14^ In our study, the calculation grid size was 1.07 × 1.07 × 2  mm for the merged images and 0.86 × 0.86 × 1.8 mm for ClearRT images, with only minor dose differences observed.

For structure volume considerations, differences between image sets impact dose‐volume outcomes. Martin et al.^11^ investigated structural volume changes when rigidly registering images with varying slice spacings for structure propagation. Their results showed the largest volume changes in small structures, such as the optic chiasm, where variations in slice spacing from 1 to 3 mm resulted in volume changes from 32.4% to 50.7%. Such variations substantially affect DVH metrics, particularly for small structures. In our study, slice thickness varied by 0.2 mm, with volume differences ranging from 0.06% to 1.41%.

In the patient study, dosimetric differences for data receiving a red notification for at least 3 consecutive days were 0.4 ± 3.0 cGy for PTV, 2.3 ± 6.0 cGy for the right parotid, and 0.9 ± 6.0 cGy for the left parotid, showing no statistically significant differences between PreciseART and TPS recalculations. The maximum percentage difference observed for red notifications was 2.28% ± 4.8% for the right parotid. Differences in patient data were influenced not only by FOV and slice spacing but also by roll correction, which accounts for positioning setup errors. PreciseART's dose recalculations included IGRT‐guided shifts in three translational directions plus roll rotation correction,^15^ whereas TPS recalculations in this study considered only translational shifts. Despite these factors, PreciseART effectively triggered alerts based on set criteria, meeting dose criteria across all notification levels: green, yellow, and red.

Several studies have investigated the optimal timing for ART in NPC. Wang et al.^16^ found that adaptive replanning before the 25^th^ fraction improved target coverage and OARs sparing. Gai et al.^17^ reported significant parotid gland volume changes within the first 4 weeks of radiation, noting that target volume reductions often exceeded 50% before the 21^st^ fraction, suggesting the need for adaptive replanning between the 21^st^ and 25^th^ fractions. Huang et al.^18^ recommended two replans: one at the 5^th^ fraction for the PTV and another at the 15^th^ fraction for both PTV and OARs. Similarly, Figen et al.^19^ identified the 15^th^ fraction as the optimal timing for replanning in head and neck cancer. Our findings support these recommendations, suggesting that adaptive replanning within the first 4 weeks benefits both PTV and OARs.

Beyond the scope of this study, four patients underwent adaptive replanning with Re‐CT simulations: two due to alerts for the right parotid and two for the left parotid. Hybrid plans were created using Re‐CT images and recontoured structures to verify PreciseART alerts based on ClearRT images. The results from these hybrid plans matched the PreciseART alerts, as shown in Table 6, suggesting that PreciseART's automated dose calculations, notifications, and deformation tracking from ClearRT images are effective when using scaled doses as a trigger tool. Notably, ClearRT images could potentially replace Re‐CT simulations for replanning, though further investigation is needed, as FOV limitations may affect adaptive planning accuracy.

**TABLE 6:** DVH metrics for red notifications by PreciseART were compared to DVH metrics from hybrid plans.

Patients	TPS (ReCT) (Gy)	PreciseART (ClearRT) (Gy)
No. 5 Rt. parotid	1.21	1.25
No. 7 Lt. parotid	1.07	1.29
No. 8 Rt. Parotid	1.26	1.13
No. 10 Lt. parotid	1.26	1.26

*Note*: The action level for both parotid glands was set at a fractional dose exceeding 1.06 Gy.

For clinical applications, Niederst et al.^20^ reported that DIR accuracy in PreciseART was insufficient in certain head and neck, thoracic, and abdominopelvic cases. Therefore, deformation accuracy should be carefully assessed, as it directly impacts dose metrics used for alerts. Clinical application of PreciseART requires thorough review and approval by a radiation oncologist.

This study is limited by a small sample size and the lack of a comprehensive analysis of deformation accuracy. Future studies with larger cohorts could provide a more comprehensive evaluation of PreciseART's dose recalculation accuracy and assess daily structure deformation accuracy and its effects on trigger tools across all treatment sites to enhance adaptive radiotherapy for HT.

## 5 CONCLUSION

This study confirms that PreciseART's daily dose recalculation is consistent with the TPS result and the notification system effectively identifies dose changes that comply with the preset dose criteria levels. PreciseART can be applied in clinical treatment to signal the need for adaptive replanning. However, PreciseART should be used with careful review and approval by a radiation oncologist due to issues related to structural contour deformation.

## AUTHOR CONTRIBUTIONS

**Yawitta Maneepan**: The conception and design; acquisition of data; analysis and interpretation of data; drafting the article. **Anirut Watcharawipha**: The conception and design; analysis and interpretation of data. **Imjai Chitapanarux**: The conception and design; analysis and interpretation of data. **Somsak Wanwilairat**: The conception and design; analysis and interpretation of data. **Wannapha Nobnop**: The conception and design; analysis and interpretation of data; revising it critically for important intellectual content and final approval of the version to be published.

## CONFLICT OF INTEREST STATEMENT

The authors declare no conflicts of interest.

## References

[1] Zhang J , Peng Y , Ding S , et al. Comparison of different combinations of irradiation mode and jaw width in helical tomotherapy for nasopharyngeal carcinoma. Front Oncol. 2020;10:598. 32391275 10.3389/fonc.2020.00598 PMC7190867

[2] Ahn PH , Chen CC , Ahn AI , et al. Adaptive planning in intensity‐modulated radiation therapy for head and neck cancers: single‐institution experience and clinical implications. Int J Radiat Oncol Biol Phys. 2011;80(3):677‐685. 20619553 10.1016/j.ijrobp.2010.03.014

[3] Glide‐Hurst CK , Lee P , Yock AD , et al. Adaptive radiation therapy (ART) strategies and technical considerations: a state of the ART review from NRG oncology. Int J Radiat Oncol Biol Phys. 2021;109(4):1054‐1075. 33470210 10.1016/j.ijrobp.2020.10.021 PMC8290862

[4] Bertholet J , Anastasi G , Noble D , et al. Patterns of practice for adaptive and real‐time radiation therapy (POP‐ART RT) part II: offline and online plan adaption for interfractional changes. Radiother Oncol. 2020;153:88‐96. 32579998 10.1016/j.radonc.2020.06.017 PMC7758781

[5] Lu S , Fan H , Hu X , et al. Dosimetric comparison of helical tomotherapy, volume‐modulated arc therapy, and fixed‐field intensity‐modulated radiation therapy in locally advanced nasopharyngeal carcinoma. Front Oncol. 2021;11:764946. 34804969 10.3389/fonc.2021.764946 PMC8602559

[6] Tegtmeier RC , Ferris WS , Bayouth JE , Miller JR , Culberson WS . Characterization of imaging performance of a novel helical kVCT for use in image‐guided and adaptive radiotherapy. J Appl Clin Med Phys. 2022;23(6):e13648. 35570390 10.1002/acm2.13648 PMC9194993

[7] Yang B , Geng H , Chang TYA , et al. Clinical implementation of kVCT‐guided tomotherapy with ClearRT. Phys Eng Sci Med. 2022;45(3):915‐924. 35925545 10.1007/s13246-022-01162-y

[8] Kainz K , Lim S , Chen GP , Li XA . PreciseART adaptive radiation therapy software: dose monitoring, re‐planning, and delivery verification. Accuray White Paper. 2017. (https://www.accuray.com/wp‐content/uploads/AP‐PreciseART_Froedtert_WP‐MKT000500.pdf)

[9] Zhong H , Garcia‐Alvarez JA , Kainz K , et al. Development of a multi‐layer quality assurance program to evaluate the uncertainty of deformable dose accumulation in adaptive radiotherapy. Med Phys. 2023;50(3):1766‐1778. 36434751 10.1002/mp.16137 PMC10033340

[10] García‐Alvarez JA , Zhong H , Schultz CJ , Li XA , Kainz K . Incorporating uncertainty bounds in daily deformable dose accumulation for adaptive radiation therapy of head‐and‐neck cancer. Med Phys. 2023;50(4):2474‐2487. 36346034 10.1002/mp.16085

[11] Martin S , KVCT YartsevS . MVCT, and hybrid CT image studies–treatment planning and dose delivery equivalence on helical tomotherapy. Med Phys. 2010;37(6):2847‐2854. 20632596 10.1118/1.3432566

[12] Zhu J , Bai T , Gu J , et al. Effects of megavoltage computed tomographic scan methodology on setup verification and adaptive dose calculation in helical TomoTherapy. Radiat Oncol. 2018;13(1):80. 29699582 10.1186/s13014-018-0989-y PMC5921977

[13] Langen KM , Meeks SL , Poole DO , et al. The use of megavoltage CT (MVCT) images for dose recomputations. Phys Med Biol. 2005;50(18):4259‐4276. 16148392 10.1088/0031-9155/50/18/002

[14] Hoshida K , Ohishi A , Mizoguchi A , Ohkura S , Kawata H . The effects of mega‐voltage CT scan parameters on offline adaptive radiation therapy. Radiol Phys Technol. 2024;17(1):248‐257. 38334889 10.1007/s12194-023-00773-8

[15] Kainz K , Garcia Alvarez J , Zhong H , et al. Use of a DVH overlay technique for quality assurance of deformable image registration‐based dose accumulation. Med Phys. 2022;49(1):611‐623. 34826153 10.1002/mp.15375

[16] Wang W , Yang H , Hu W , et al. Clinical study of the necessity of replanning before the 25th fraction during the course of intensity‐modulated radiotherapy for patients with nasopharyngeal carcinoma. Int J Radiat Oncol Biol Phys. 2010;77(2):617‐621. 20138444 10.1016/j.ijrobp.2009.08.036

[17] Gai X , Wei Y , Tao H , Zhu J , Li B . Clinical study of the time of repeated computed tomography and replanning for patients with nasopharyngeal carcinoma. Oncotarget. 2017;8(16):27529‐27540. 28404877 10.18632/oncotarget.16770 PMC5432355

[18] Huang H , Lu H , Feng G , et al. Determining appropriate timing of adaptive radiation therapy for nasopharyngeal carcinoma during intensity‐modulated radiation therapy. Radiat Oncol. 2015;10:192. 26377685 10.1186/s13014-015-0498-1 PMC4573680

[19] Figen M , Colpan Oksuz D , Duman E , et al. Radiotherapy for head and neck cancer: evaluation of triggered adaptive replanning in routine practice. Front Oncol. 2020;10:579917. 33282734 10.3389/fonc.2020.579917 PMC7690320

[20] Niederst C , Dehaynin N , Lallement A , Meyer P . Image processing pitfalls in vendor adaptive radiotherapy software with tomotherapy‐like systems: feedback from clinical case reports. Curr Med Imaging. 2023;19(10):1156‐1166. 36631921 10.2174/1573405619666230111114244

